# Fungal Inhibition of Agricultural Soil Pathogen Stimulated by Nitrogen-Reducing Fertilization

**DOI:** 10.3389/fbioe.2022.866419

**Published:** 2022-04-12

**Authors:** Min-Chong Shen, You-Zhi Shi, Guo-Dong Bo, Xin-Min Liu

**Affiliations:** ^1^ Tobacco Research Institute of Chinese Academy of Agricultural Sciences, Qingdao, China; ^2^ Cigar Institute of China Tobacco Hubei Industrial Co., Ltd., Yichang, China

**Keywords:** nitrogen-reducing fertilization, variation of fungal community, fungal inhibition, cash crop, sustainable agriculture

## Abstract

Plant health is the fundamental of agricultural production, which is threatened by plant pathogens severely. The previous studies exhibited the effects of different pathogen control strategies (physical, chemical, and microbial methods), which resulted from bringing in exogenous additives, on microbial community structures and functions. Nevertheless, few studies focused on the potential inhibitory abilities of native microbial community in the soil, which could be activated or enhanced by different fertilization strategies. In this study, three plant diseases (TMV, TBS, and TBW) of tobacco, fungal community of tobacco rhizosphere soil, and the correlation between them were researched. The results showed that nitrogen-reducing fertilization strategies could significantly decrease the occurrence rate and the disease index of three tobacco diseases. The results of bioinformatics analyses revealed that the fungal communities of different treatments could differentiate the nitrogen-reducing fertilization group and the control group (CK). Furthermore, key genera which were responsible for the variation of fungal community were explored by LEfSe analysis. For instance, *Tausonia* and *Trichocladium* increased, while *Naganishia* and *Fusicolla* decreased under nitrogen-reducing fertilization conditions. Additionally, the correlation between tobacco diseases and key genera was verified using the Mantel test. Moreover, the causal relationship between key genera and tobacco diseases was deeply explored by PLS–PM analysis. These findings provide a theoretical basis for a nitrogen-reducing fertilization strategy against tobacco diseases without exogenous additives and make contributions to revealing the microbial mechanism of native-valued fungal key taxa against tobacco diseases, which could be stimulated by agricultural fertilization management.

## Introduction

Plant diseases can devastate agriculture by reducing crop yields and causing severe economic losses (Strange and Scott, 2005). Plant diseases caused by soilborne pathogens can diminish the yields of vegetables, fruits, and other economically important crops by up to 20% (Jaiswal et al., 2018). The perniciousness of plant diseases has risen significantly due to current agricultural practices including intensive cultivation, overuse of fertilizers, utilization of high-yield but pathogen-susceptible cultivars, and consecutive years of continuous cropping (Chakraborty and Newton, 2011; Ghorbanpour et al., 2018). Thus, disease management strategies targeting plant pathogens are of vital importance (Peng et al., 2017).

Much effort has been expended in exploring disease management strategies under various circumstances. Physical methods such as heating and solarizing the soil are laborious, and they can hardly eradicate pathogens (De Carvalho Pontes et al., 2019). Chemical methods such as pesticides are expensive, they can poison the environment, and they can result in pesticide-resistant bacterial populations (Morais et al., 2019) that may become ineffective in the face of the bacterial genetic variability (Kim et al., 2016). Crop rotation is a slow method for reducing pathogen density in soil, making it unsuitable when severe disease breakout (Chellemi et al., 2016). Biocontrol bacteria are receiving increasing attention as a potential alternative to traditional methods for controlling plant diseases (Choi et al., 2020; Ogunnaike et al., 2021; Ahmed et al., 2022), based on being harmless, effective, and sustainable (Xie et al., 2020). Previous studies have identified various biocontrol agents including *Bacillus* spp. (Cao et al., 2011), *Paenibacillus polymyxa* (Chunyu et al., 2017), *Streptomyces* spp. (Abo-Zaid et al., 2020), and *Pseudomonas fluorescens* (Huang et al., 2020). Additionally, a non-native arbuscular mycorrhizal fungal inoculant has been reported to improve the growth and enhance the resistance system of multiple plants (Pellegrino et al., 2012; Aliyu et al., 2019; Yang L et al., 2021).

Previous research corresponding to physical, chemical, and microbial strategies against plant diseases discovered that most of the strategies could convert the microbial community of the rhizosphere soil of plants into an enhanced status of pathogen resistance (Hu et al., 2020; Liu et al., 2020). According to the principle of low cost and environmental friendliness, strategies without exogenous additives received more and more attention. Evidence had demonstrated that nitrogen-reducing fertilization could change the microbial community of planting soils (Zhou et al., 2020; Yang T et al., 2021). As a no-additive strategy, the inhibitory effect of nitrogen-reducing fertilization against tobacco diseases was little researched.

In this study, a nitrogen-reducing fertilization strategy was used in the process of tobacco planting. The occurrence rate and disease index of tobacco diseases, including tobacco mosaic virus (TMV), tobacco black shank (TBS), and tobacco bacterial wilt (TBW), were investigated. The fungal community of tobacco rhizosphere soil differentiated the nitrogen-reducing treatments from the control group (CK). The key taxa which contributed to the variation of fungal community due to nitrogen-reducing fertilization were analyzed and identified. Moreover, the dominant role played by the key taxa to help inhibit tobacco diseases was verified using the Mantel test. This study contributed to reveal the conversion of the fungal community against tobacco pathogens, which provided a feasible, economic, and eco-friendly fertilization strategy in the tobacco-planting industry.

## Materials and Methods

### Design of Field Experiments

This experiment was conducted in Huangdao district, Qingdao, Shandong Province (36°00′44.86″ N, 119°51′35.30″ E), China, from April 10 to 15 October 2020. The average annual rainfall of the experimental field was 696.6 mm, the annual sunshine duration was 2,110.1 h, and the annual average temperature was 12.5 C. The previous crop in the experiment site was tobacco (Zhongyan 100 variety), which had been planted for four consecutive years. The flue-cured tobacco variety used in this experiment was Zhongyan 100. The fertilization procedure in this experiment was carried out according to the fertilization strategies (Supplementary Table S1). The tobacco was transplanted at May 5, and tobacco diseases were investigated on July 23, according to the grade and investigation method of tobacco diseases and insect pests (Tobacco Research Institute, 2008).

### Collection and Preprocessing of Soil Samples

Rhizosphere soil is the soil that lies much closer to the plant roots. The samples of tobacco rhizosphere soil were collected using a sterilized brush and by gentle shaking (Pétriacq et al., 2017). Three replicates were prepared for each treatment. The soil samples were immediately stored in a −20°C refrigerator for subsequent extraction of soil DNA.

### DNA Extraction and Amplicon Sequencing of ITS Genes

About 500 mg soil sample was collected from well-mixed rhizosphere soil from each replication. DNA was extracted from the soil using a DNA extraction kit (FastDNA^**TM**^ SPIN Kit for soil, MP Biomedicals, LLC, Solon, OH, United States) according to the manufacturer’s instructions. Subsequently, DNA was tested with 1% agarose gel, and the successfully extracted DNA was stored at −20°C immediately.

The PCR of the ITS1 region of the fungal ITS gene sequencing was conducted using the specific primers CTT​GGT​CAT​TTA​GAG​GAA​GTA​A and GCT​GCG​TTC​TTC​ATC​GAT​GC. The amplification products were tested for specificity in 1% agarose gel (Yuan et al., 2020). Then, the library was constructed using the library construction kit TruSeq^®^ DNA PCR-Free Sample Preparation kit (Illumina, San Diego, CA, United States). After the library was successfully constructed, the quantitative process was implemented using the Qubit^®^ 2.0 Fluorometer (Life Technologies, Carlsbad, CA, United States) and qPCR. After the quantitative test, subsequent sequencing was performed on the Illumina MiSeq platform.

### Processing and Analyses of Bioinformatics and Tobacco Disease Data

The raw data after sequencing were assembled and attached by FLASH (V1.2.11, https://ccb.jhu.edu/software/FLASH/index.shtml) to obtain raw tags. Subsequently, QIIME software (V1.9.1, http://qiime.org/scripts/split_libraries_fastq.html) was used to filter raw tags. Then, the UCHIME algorithm (http://www.drive5.com/usearch/manual/uchime_algo.html) (Haas et al., 2011) was used to detect and remove chimeras against the Unite database (https://unite.ut.ee/) (Edgar et al., 2011) and get effective data (effective tags) (Callahan et al., 2016).

The valid data from all samples were clustered into the same operational taxonomic units (OTUs), and the OTUs with the highest frequency were selected as the representative of OTU sequences. The ITS database Unite (https://unite.ut.ee/) for species annotation analysis was used subsequently (Kõljalg et al., 2013). Finally, all OTUs were uniformized based on the smallest amount of sequencing data as the standard.

Afterward, the normalization process was followed by analyses performed based on the OTU statistics. Qiime software (version 1.9.1, http://qiime.org/install/index.html) was used to calculate Shannon, Simpson, Ace, Chao1, and PD whole tree indexes, and R software (version 3.6.0) was used to draw a rarefaction curve. Then, the Bray–Curtis distance was calculated by Qiime software (version 1.9.1), and the WGCNA, stats, and ggplot2 packages of R software (version 3.6.0) were used to draw PCA, PCoA, NMDS, and PLS-DA plots. The vegan package based on R software (version 3.6.0) was used to test the differences in the microbial community structure among different treatments through PERMANOVA. Subsequently, the significance *P*-value in the LEfSe analysis was conducted using the Wilcoxon rank-sum test. The LDA was set to 3.0. The correlation between fungal data and tobacco disease data was calculated, according to the Mantel test (Li Y. et al., 2021). Based on the analysis platform of Majorbio (Shanghai Majorbio Bio-pharm Technology Co., Ltd., Shanghai, China), the results of species annotations were used for further comparative analyses. The raw sequence data were uploaded to the Sequence Read Archive (accession number: PRJNA808161), which could be freely available on the NCBI (https://www.ncbi.nlm.nih.gov/bioproject/PRJNA808161).

The tobacco diseases data were analyzed by SPSS (version 25.0), and the single-factor ANOVA (Duncan’s multiple range test) in SPSS software was used to calculate the significance of the differences among nitrogen-reducing fertilization and CK. All figures were drawn through Origin (version 2018) (OriginLab Corporation, Northampton, MA, United States) and R software (version 3.6.0). All results were presented as mean ± standard deviation.

## Results

### Nitrogen-Reducing Fertilization Impacted the Occurrence and Severity of Tobacco Diseases

Regarded as the main plant diseases of tobacco, tobacco mosaic virus (TMV), tobacco black shank (TBS), and tobacco bacterial wilt (TBW) were investigated. The disease occurrence rates and disease indexes of the three diseases were calculated among all treatments (Table 1). The results demonstrated that nitrogen-reducing fertilization could decrease the occurrence rate and the disease index of tobacco diseases. In particular, all three nitrogen-reducing fertilization strategies (RNTe, RNTw, and RNTh) reduced the occurrence rate of TMV and TBS significantly (*p* < 0.05), compared to CK. Although the inhibitory effects of nitrogen-reducing fertilization strategies were weakened when they were faced by TBW, the difference of the disease occurrence rate between RNTe and CK reached the significant level (*p* < 0.05). Furthermore, the tendency of the disease index of these three tobacco diseases was consistent with the principle of their occurrence rates, except that there were no significant differences between RNTh and CK in the disease index of TBS.

**TABLE 1:** Occurrence rate and index of tobacco diseases in different treatments.

Treatment	Disease occurrence rate/%			Disease index		
	TMV	TBS	TBW	TMV	TBS	TBW
CK	32.74 ± 0.91d	20.93 ± 1.09d	33.02 ± 1.30b	6.91 ± 0.13d	5.41 ± 0.08c	9.57 ± 0.18b
RNTe	16.38 ± 0.80a	11.29 ± 1.16a	28.11 ± 1.36a	5.05 ± 0.20a	4.07 ± 0.13a	9.14 ± 0.15a
RNTw	24.15 ± 0.96b	16.13 ± 0.32b	29.80 ± 1.59ab	5.67 ± 0.09b	4.88 ± 0.08b	9.27 ± 0.10ab
RNTh	28.06 ± 1.21c	18.62 ± 1.26c	31.47 ± 1.61ab	6.32 ± 0.22c	5.27 ± 0.09c	9.41 ± 0.12ab

TMV: tobacco mosaic virus; TBS: tobacco black shank; TBW: tobacco bacterial wilt. All data in the table are presented as means ± standard deviation (SD). Means followed by different lowercase letters are significantly different at the 5% level by DMRT (Duncan’s multiple range test).

### Nitrogen-Reducing Fertilization Impacted the Structure of Fungal Communities

**FIGURE 1:**
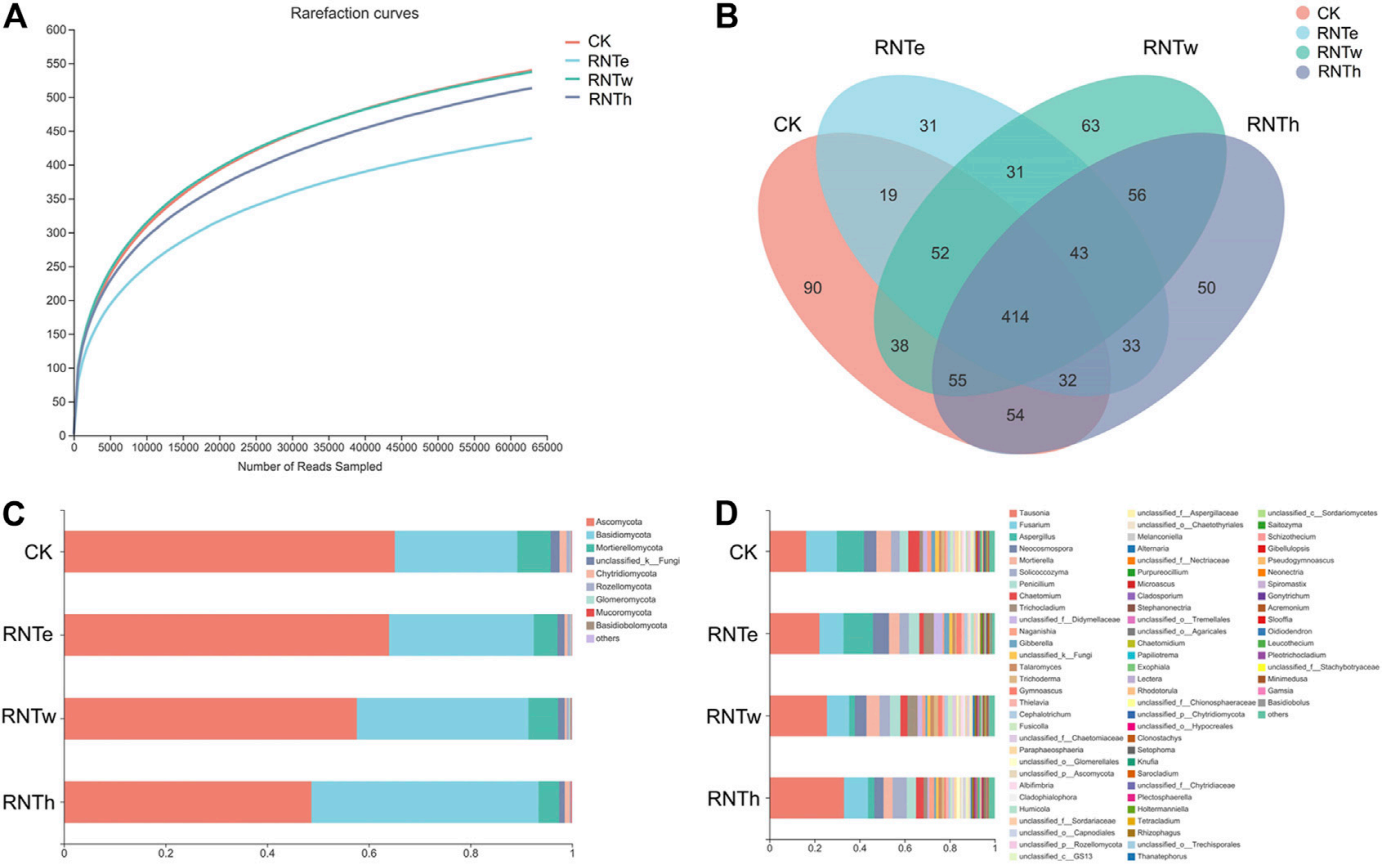
Composition of fungal communities in different treatments, based on OTU level. **(A)** Rarefaction curves of different treatments. **(B)** Venn diagram of different treatments, based on the number of OTUs. **(C)** Main taxa of fungi in different treatments at the phylum level. **(D)** Main taxa of fungi in different treatments at the genus level.

To explore the microbial mechanism of the inhibitory effect of nitrogen-reducing fertilization strategies on tobacco diseases, the fungal community of different treatments was further researched. The composition of the fungal community was influenced by nitrogen-reducing fertilization (Figure 1 and Supplementary Figure S1). The lowest rarefaction curve occurred in RNTe, which represented that the richness of fungal community was smallest in RNTe, compared to other treatments (Figure 1A). The Venn diagram indicated that 754, 655, 752, and 737 OTUs were observed in CK, RNTe, RNTw, and RNTh, respectively. Among them, all treatments shared 414 common OTUs, whereas CK, RNTe, RNTw, and RNTh owned 90, 31, 63, and 50 unique OTUs, respectively (Figure 1B). According to the results of annotation, Ascomycota, Basidiomycota, and Mortierellomycota were the dominant fungi based on the phylum level. Due to nitrogen-reducing fertilization, the relative abundance of Ascomycota was decreased, whereas the relative abundance of Basidiomycota was improved. Additionally, the relative abundance of Mortierellomycota was up and down among different treatments, without an apparent discipline (Figure 1C). Corresponding to the genus level, the relative abundance of *Tausonia* was increased by nitrogen-reducing fertilization, while that of more genera (such as *Fusarium* and *Aspergillus*) was declined (Figure 1D).

**FIGURE 2:**
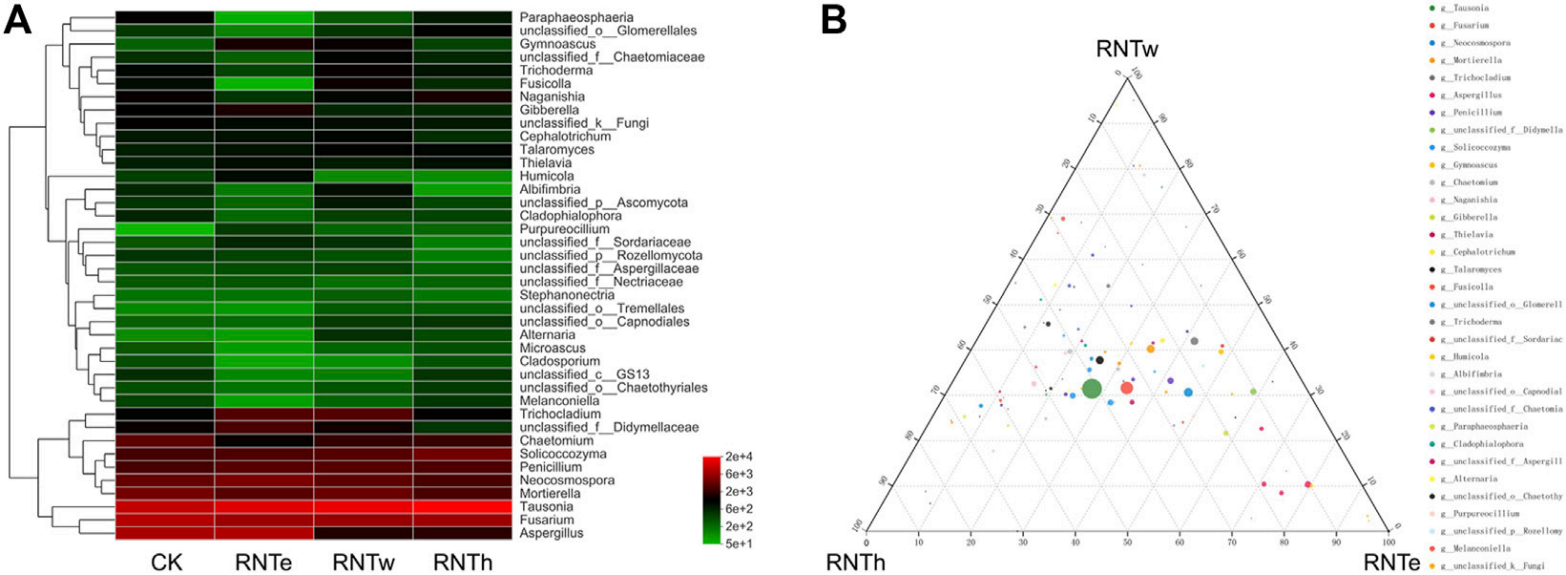
Composition of fungal communities in different treatments, based on the genus level. **(A)** Heat map of top 40 genera of different treatments. **(B)** Ternary analysis of different treatments, based on the genus level.

Based on the heat map analysis, compared with the control, nitrogen reduction treatment increased the abundance of some genera and decreased the abundance of other genera (Figure 2A). In particular, nitrogen reduction treatment showed a strong trend of decreasing the relative abundance of genera for an increase in the relative abundance of certain bacterial genera. Based on the ternary analysis, the distribution of the main dominant bacterial genera (based on relative abundance) was relatively uniform among different nitrogen reduction treatments (Figure 2B). It demonstrated the common effect of different nitrogen reduction treatments on soil fungal communities. In addition, compared with RNTh, more species of the genera were enriched in RNTe and RNTw treatment, especially RNTw.

**FIGURE 3:**
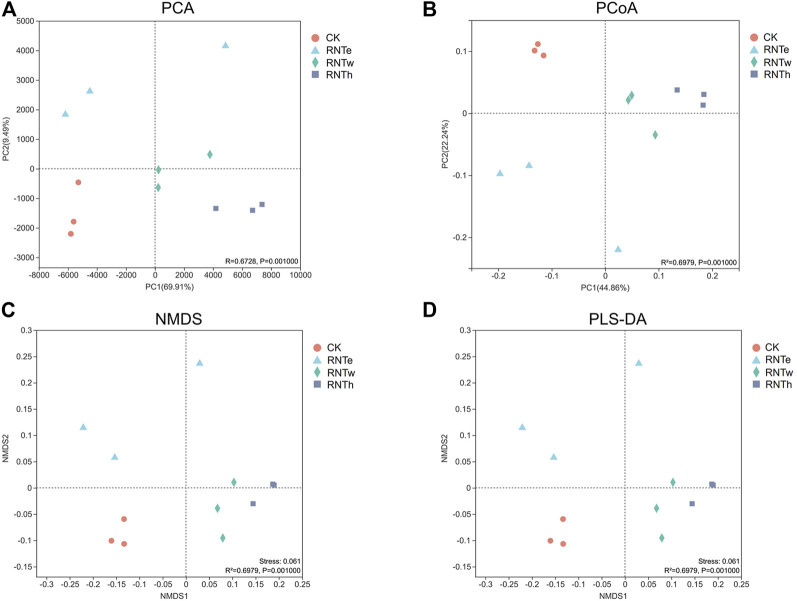
Combined plots of PCA **(A)**, PCoA **(B)**, NMDS **(C)**, and PLS-DA **(D)** of fungal communities in different treatments.

In different analyses, the fungal communities of the CK treatment were clustered into a unique area, revealing that the fungal community structure of the nitrogen-reducing treatment was significantly different from that of CK (Figure 3). In addition, the fungal communities of RNTw and RNTh clustered into a much closer location, which indicated that the community structure of the two was more similar.

### Nitrogen-Reducing Fertilization Drove the Differentiation of Key Taxa Among Different Treatments

**FIGURE 4:**
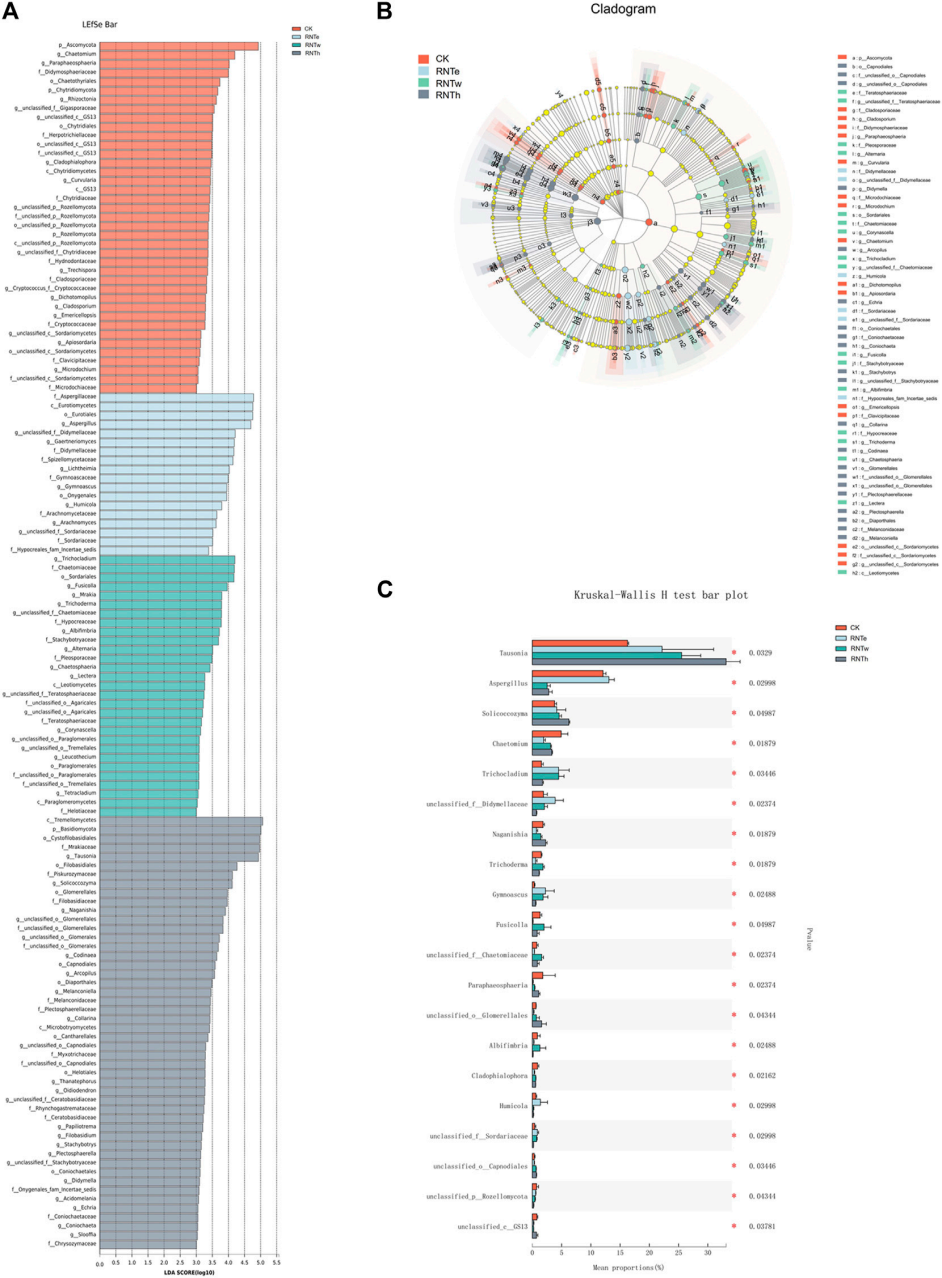
Combined plots of exploring key taxa among different treatments. **(A)** LDA effect size (LEfSe) analysis based on the genus among different treatments. **(B)** Cladogram of biomarkers among different treatments. **(C)** Bar plot of top 20 significant differential taxa based on the Kruskal–Wallis H test.

Furthermore, the LEfSe analysis of different treatments revealed that biomarkers of different treatments were identified as 4 phyla and 64 genera, among which CK, RNTe, RNTw, and RNTh had 17, 8, 16, and 23 genera, respectively (Figures 4A,B). Based on the relative abundance, the top 20 genera with significant differences among different treatments were further analyzed, revealing the key genera that caused the changes in the fungal community structure in the nitrogen reduction treatment, compared to CK (Figure 4C). Specifically, the relative abundance of *Tausonia*, *Trichocladium*, unclassified *Didymellaceae*, *Gymnoascus*, *Humicola*, and unclassified *Sordariaceae* was significantly (*p* < 0.05) enhanced by nitrogen-reducing fertilization, whereas the relative abundance of *Chaetomium*, *Naganishia*, *Trichoderma*, *Fusicolla*, *Paraphaeosphaeria*, *Albifimbria*, *Cladophialophora*, and unclassified *GS13* was significantly declined by nitrogen-reducing fertilization (*p* < 0.05).

**FIGURE 5:**
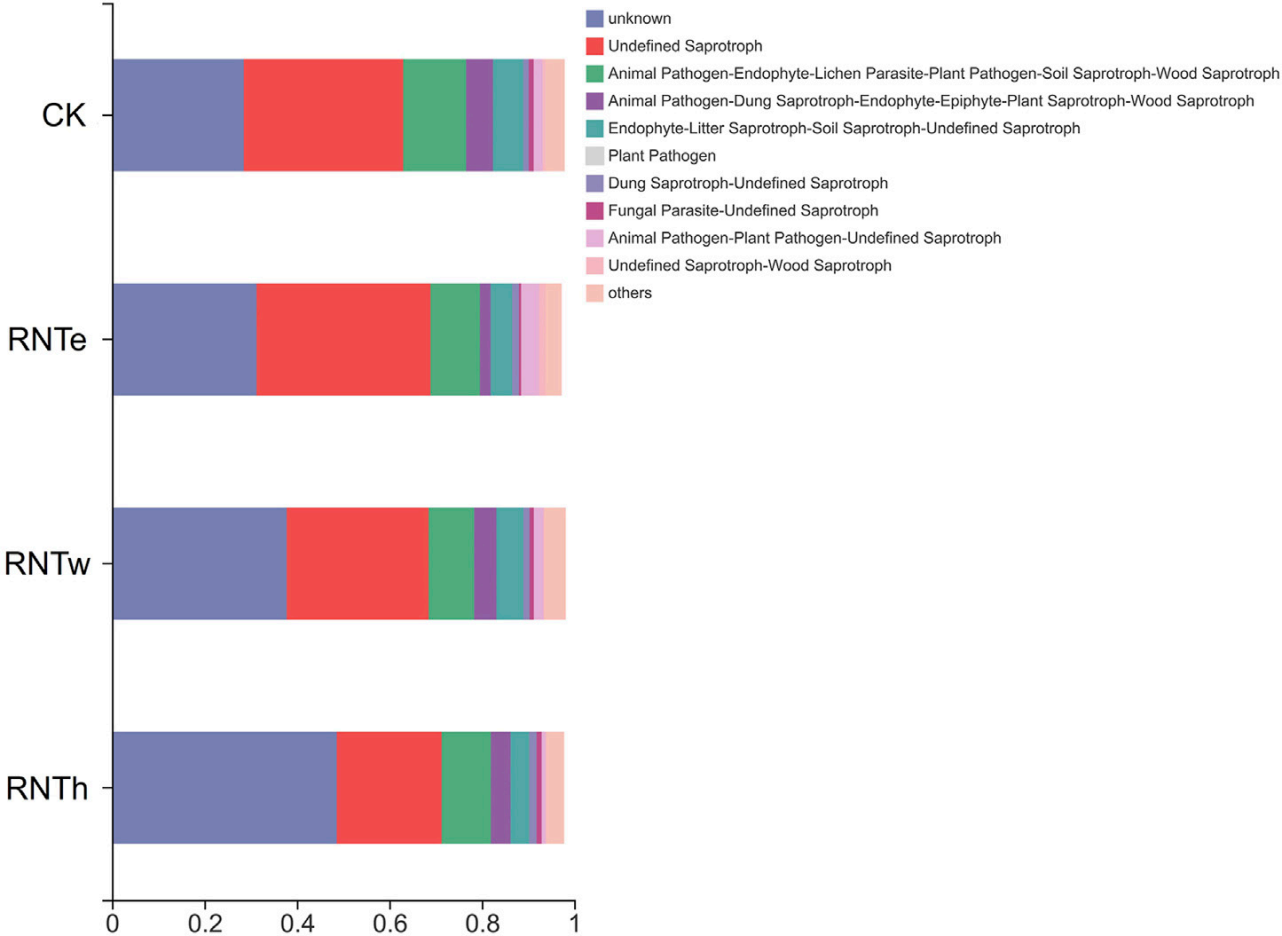
Predictive functions of fungal communities of different treatments.

Furthermore, the fungal community functions of different treatments were predicted (Figure 5). In this study, different types of saprophytic functions are the dominant functions of fungal communities in different treatments. Compared with the control, nitrogen reduction treatment reduced the pathological nutritional functions of animals and plants and enhanced their saprophytic nutritional functions. In addition, nitrogen reduction treatments significantly enhanced unknown functions of the fungal community. The dominant effect of nitrogen reduction treatment in the process of tobacco growing is not only derived from its changes in common functions but also related to its activation of new and unknown functions.

### Correlation Between Fungal Key Taxa and Tobacco Diseases

To verify the microbial mechanism of the inhibitory effect against tobacco diseases caused by nitrogen-reducing fertilization, the Mantel test was implemented between key taxa and the results of tobacco diseases (Table 2). The results of the Mantel test indicated that the key taxa, whose relative abundances were significantly (*p* < 0.05) different from CK, had strong correlations to the occurrence rates and disease indexes of tobacco diseases among different treatments. Interestingly, the correlations between fungal key taxa were much stronger in inhibition against TMV and TBS with the R values (occurrence rate) of 0.7733 and 0.6927, and the R values (disease index) of 0.7194 and 0.7197, rather than in the inhibition against TBW.

**TABLE 2:** Mantel test of correlations between key taxa and the results of tobacco diseases.

		Key taxa (CK-RNTe)	
		R value	P value
Occurrence rate	TMV	0.7733	0.001
	TBS	0.6927	0.001
	TBW	0.3658	0.021
Disease index	TMV	0.7194	0.001
	TBS	0.7197	0.001
	TBW	0.3281	0.048

Key taxa refer to those that are significantly different from CK, based on OTU statistics.

**FIGURE 6:**
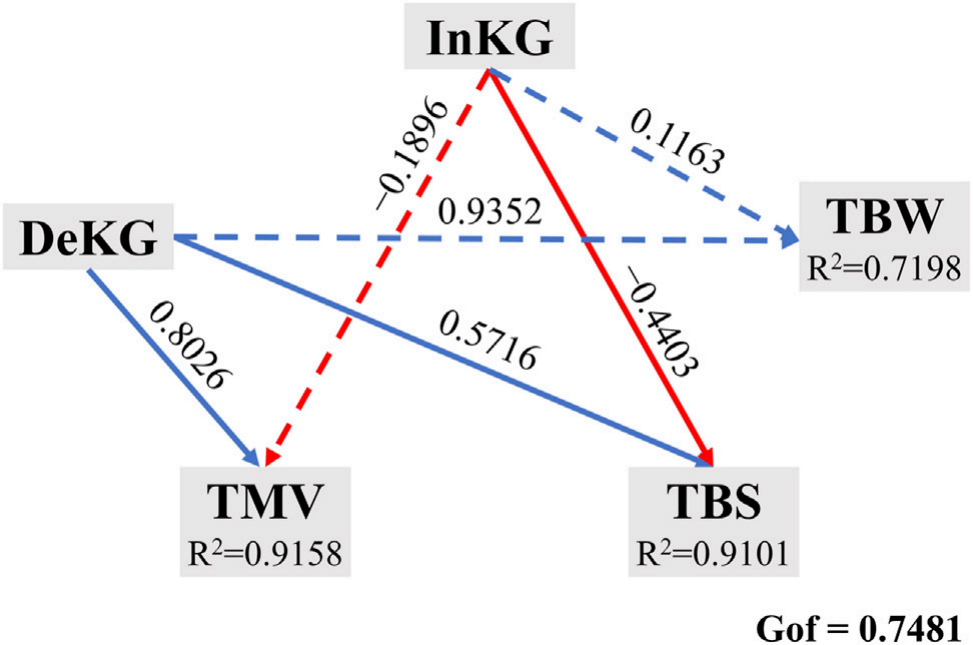
PLS-PM diagram based on the path coefficients between different modules. Blue and red arrow lines represent the positive and negative causal relationship between each two modules, respectively. The lines composed of dash lines means the calculated result of the relationship between the two modules did not reach the significant (*p* < 0.05) level. InKG: the key genera whose relative abundances were significantly increased by nitrogen-reducing fertilization. DeKG: the key genera whose relative abundances were significantly decreased by nitrogen-reducing fertilization. Gof: goodness of fit. *R* ^2^ indicated the amount of variance in the endogenous latent variable explained by its independent latent variables.

To deeply pursue the causal relationship between key genera and those three tobacco diseases, partial least squares path modeling (PLS-PM) was employed to analyze the paths among all the modules (Figure 6). Five modules, the key genera whose relative abundances were significantly increased by nitrogen-reducing fertilization (InKG), the key genera whose relative abundances were significantly decreased by nitrogen-reducing fertilization (DeKG), TMV, TBS, and TBW, were set to establish an analysis model. The goodness of fit (Gof) was 0.7481, which meant the prediction power of the model was of 74.81%. The results of path coefficients showed that InKG had a negative causal relationship with TMV and TBS and had a positive causal relationship with TBW, whereas DeKG had a positive causal relationship with those three tobacco diseases. However, among these path coefficients, the causal relationships between InKG, TMV, and TBW were not significant (*p* < 0.05). Additionally, the causal relationship between DeKG and TBW did not reach a significant (*p* < 0.05) level neither. Furthermore, the *R* ^2^ values of TMV, TBS, and TBW, which represented the amount of variance of the three diseases explained by this model, were 0.9158, 0.9101, and 0.7198, respectively.

## Discussion

Plant health is the fundamental of agricultural production, which is threatened by plant pathogens severely. Regarded as one of the most popular commodities, merchandise corresponding to tobacco plays an important role in undertaking the national economic task. However, the tobacco-planting industry is hindered by plant diseases such as tobacco mosaic virus, tobacco black shrank, and tobacco bacterial wilt (Hu Q. et al., 2021; Hu Y. et al., 2021). Chemicals, chemical pesticide, soil fumigation, and microbial inoculants consist of the mainstream pathogen control strategies that have earned great achievements fighting against plant diseases (Alori and Babalola, 2018; Ren et al., 2018; Raymaekers et al., 2020; Long et al., 2021). The previous studies exhibited the effects of different pathogen control strategies, which resulted from exogenous additives, on microbial community structures and functions (Preininger et al., 2018; Li et al., 2020; Vassilev et al., 2020). Nevertheless, without additives, the soil microbial structure could be changed according to different amounts of fertilizers (Li B.-B. et al., 2021). It would be of great help to the agricultural management which could prevent cash crops from plant diseases using a fertilization strategy.

In this study, nitrogen-reducing fertilization strategies showed an obvious inhibitory effect against tobacco diseases, especially against TMV and TBS. Through the composition and structure of fungal communities between nitrogen-reducing fertilization and CK, the variation of fungal communities was explored to exhibit the effects of nitrogen-reducing fertilization on converting the microbial community into an inhibition-enhanced status. Previous studies had indicated that the appropriate reduction of chemical fertilizers, especially nitrogen fertilizer, could improve the yield of crops and impact the microbial community in rhizosphere soil of plants. Some research studies paid attention to the effects of the nitrogen-reducing strategy on fungal community of food crops such as wheat, rice, and maize (Wen et al., 2020; Liu et al., 2021; Wang et al., 2021). Wang et al. found that *Humicola*, *Tausonia*, and *Codinaea* could improve the chemical quality of tobacco (Wang et al., 2022). Nevertheless, few had focused on the fungal inhibitory effect of nitrogen-reducing fertilization on tobacco diseases.

Interestingly, the bioinformatics of the fungal community of all treatments were found to be closely correlated to the results of tobacco diseases. Based on LEfSe analysis and Kruskal–Wallis H test, the key taxa that dominated the variation of fungal communities between nitrogen-reducing fertilization and CK were excavated and summarized. Previous evidence verified that those abundance-improved genera, which were enhanced by nitrogen-reducing fertilization, were more likely to benefit the plants and their soil environment. For instance, *Tausonia* could help plants to tolerate low temperature and improve the fermentation process (Kim et al., 2020). *Trichocladium* was verified to be used as biofertilizers and biofungicides, which played an important role in declining the tobacco disease occurrence rate and index. Furthermore, some species of *Trichocladium* could produce cellulolytic enzymes, which provided microorganisms with carbon sources and provided tobacco with nutrients (Kubicek et al., 2019). Meanwhile, nitrogen-reducing fertilization not only enhanced the beneficial fungi in tobacco rhizosphere soil but also decreased the negative. For instance, *Naganishia* (Horváth et al., 2020), *Trichoderma* (Poveda et al., 2020), *Fusicolla* (Clocchiatti et al., 2021), *Paraphaeosphaeria* (Ding et al., 2020), and *Albifimbria* (Matić et al., 2019) were reported to be plant pathogens that destroyed the growth and yield of many crops (Masachis et al., 2016).

Afterward, the correlation between key taxa and tobacco diseases was analyzed, based on the Mantel test (Crabot et al., 2019). The results indicated that the key taxa, whose relative abundances were significantly (*p* < 0.05) different from CK, made great contributions to the present variation of occurrence rates and disease indexes of tobacco diseases among different treatments. It proved that the key taxa excavated in this study were the dominant reason why nitrogen-reducing fertilization could decrease the occurrence rate and disease index of tobacco diseases. Although the results of correlation between key taxa and tobacco diseases emphasized the importance of the key genera, the causal relationships between these genera and tobacco diseases were beyond understanding. Hence, the PLS-PM analysis was implemented to address this issue (Sanchez, 2013). Through the results, the operating mechanisms of key genera, which were integrated into two modules (InKG and DeKG), in the performance of inhibiting tobacco diseases were revealed. From the aspect of TMV, the inhibitory effect of nitrogen-reducing fertilization against TMV primarily owed to the decreased key genera which caused by this strategy. When it came to TBS, the increased key genera and decreased key genera both made contributions to the inhibitory effect of this strategy, whereas InKG and DeKG did not have significant path coefficients with TBW, which indicated that there was no obvious causal relationship between key genera and TBW. The reason why RNTe could decline the TBW should be further explored beyond the fungal community of rhizosphere soil. The endogenous fungus of tobacco was suggested to be researched.

Moreover, the inhibitory effect of nitrogen-reducing fertilization was highest in RNTe, while the efficiency declined from RNTe to RNTh. It demonstrated that the reducing amount of nitrogen fertilizer was limited according to the performance of this strategy. In the present research, ten percentage of nitrogen-reducing fertilization was appropriate for tobacco planting against diseases. Additionally, the inhibitory effect of nitrogen-reducing fertilization against TBW was much lower than that against TMV and TBS. Other methods should be cooperated with this fertilization strategy to better protect tobacco from TBW.

## Conclusion

In this study, the inhibitory effect of nitrogen-reducing fertilization strategy against tobacco diseases was investigated to be significantly effective in tobacco-planting agriculture. The best performance of disease control occurred in 10% reduction of nitrogen fertilizer. The variation of the fungal community between nitrogen-reducing fertilization and CK was highly consistent with the circumstances of tobacco diseases. Furthermore, the positive genera such as *Tausonia* and *Trichocladium* and the negative genera such as *Naganishia* and *Fusicolla* were identified to be the key genera which dominated the fungal inhibition against tobacco diseases, due to the economic and eco-friendly fertilization strategy. This study provides a constructive strategy to inhibit tobacco diseases in continuous cropping areas of tobacco. It also provides a theoretical basis for developing the potential fungal inhibitory effects of native microorganisms stimulated by nitrogen-reducing fertilization.

## Data Availability

The data presented in the study are deposited in the NCBI repository (https://www.ncbi.nlm.nih.gov/bioproject/PRJNA808161), accession number: PRJNA808161.
