# Supplementary figures and images for: Fungal Inhibition of Agricultural Soil Pathogen Stimulated by Nitrogen-Reducing Fertilization
Source: Front Bioeng Biotechnol. 2022 Apr 12;10:866419. doi: 10.3389/fbioe.2022.866419 (PMC9039341; doi:10.3389/fbioe.2022.866419)

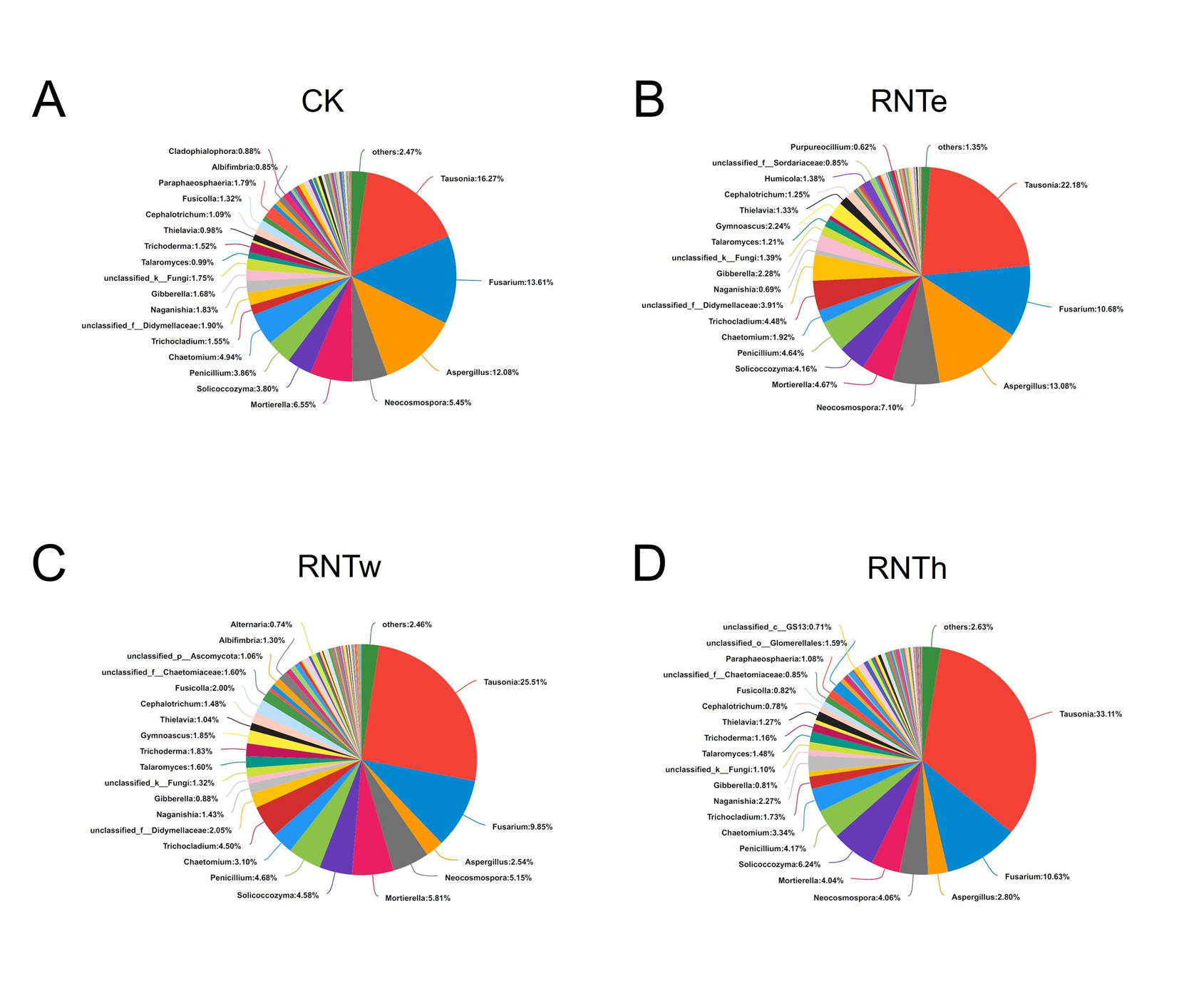

Supplement: Supplementary file 1 [file Image1.JPEG]
