# Supplementary material for: Fungal Inhibition of Agricultural Soil Pathogen Stimulated by Nitrogen-Reducing Fertilization
Source: Front Bioeng Biotechnol. 2022 Apr 12;10:866419. doi: 10.3389/fbioe.2022.866419 (PMC9039341; doi:10.3389/fbioe.2022.866419)
Supplement: Supplementary file 2 [file DataSheet1.docx]

***Supplementary Materials***

**Fungal Inhibition of Agricultural Soil Pathogen Stimulated by Nitrogen-Reducing Fertilization**

**Min-Chong Shen^1^, You-Zhi Shi^2^, Guo-Dong Bo^1^, and Xin-Min Liu^1*^**

^1^ Tobacco Research Institute of Chinese Academy of Agricultural Sciences, Qingdao 266101, China

^2^ Cigar Institute of China Tobacco Hubei Industrial Co., Ltd., Yichang 443100, China

*** Correspondence:**Xin-Min Liu
liuxinmin@caas.cn

**Keywords: nitrogen-reducing fertilization; variation of fungal community; fungal inhibition; cash crop; sustainable agriculture**

**Table S1.** Fertilization strategies of field experiments.

| **Treatments** | **Fertilization Strategies** |
| --- | --- |
| CK | Fermented soybeans (N: P: K= 6: 1: 2), 300 kg ha^-1^; Tobacco fomulated fertilizer (N: P: K= 10: 10: 20), 324 kg ha^-1^; Diammonium Phosphate (N: P: K= 18: 46: 0), 30 kg ha^-1^; Potassium sulfate (N: P: K= 0: 0: 50), 153 kg ha^-1^. |
| RNTe | Fermented soybeans (N: P: K= 6: 1: 2), 270 kg ha^-1^; Tobacco fomulated fertilizer (N: P: K= 10: 10: 20), 291.6 kg ha^-1^; Diammonium Phosphate (N: P: K= 18: 46: 0), 27 kg ha^-1^; Potassium sulfate (N: P: K= 0: 0: 50), 167.16 kg ha^-1^; Calcium Superphosphate (N: P: K= 0: 20: 0), 24.6 kg ha^-1^. |
| RNTw | Fermented soybeans (N: P: K= 6: 1: 2), 240 kg ha^-1^; Tobacco fomulated fertilizer (N: P: K= 10: 10: 20), 259.2 kg ha^-1^; Diammonium Phosphate (N: P: K= 18: 46: 0), 24 kg ha^-1^; Potassium sulfate (N: P: K= 0: 0: 50), 181.32 kg ha^-1^; Calcium Superphosphate (N: P: K= 0: 20: 0), 49.2 kg ha^-1^. |
| RNTh | Fermented soybeans (N: P: K= 6: 1: 2), 210 kg ha^-1^; Tobacco fomulated fertilizer (N: P: K= 10: 10: 20), 226.8 kg ha^-1^; Diammonium Phosphate (N: P: K= 18: 46: 0), 21 kg ha^-1^; Potassium sulfate (N: P: K= 0: 0: 50), 195.48 kg ha^-1^; Calcium Superphosphate (N: P: K= 0: 20: 0), 73.8 kg ha^-1^. |

**Table S2.** **The physicochemical data of the tobacco-planting soil.**

| **Soil type** | **pH** | **AN/ mg kg^-1^** | **AP/ mg kg^-1^** | **AK/ mg kg^-1^** | **SOM/ g kg^-1^** | **AMg/ mg kg^-1^** | **AZn/ mg kg^-1^** | **AB/ mg kg^-1^** | **AMo/ mg kg^-1^** |
| --- | --- | --- | --- | --- | --- | --- | --- | --- | --- |
| Brown soil | 6.09 | 105.55 | 23.01 | 105.68 | 10.02 | 277.30 | 1.14 | 0.68 | 0.043 |

AN represented available nitrogen; AP represented available phosphorus; AK represented available potassium; SOM represented soil organic matter; AMg represented available magnesium; AZn represented available zinc; AB represented available boron; Amo represented available molybdenum.

**Table S3. The OTU table of all treatments.**

**Note:** As the Table S3 was too large to be placed in this Word, please check the Excel file named “Table S3: The OTU table of all treatments.”
